# Exploring Tobacco and E-Cigarette Use among Queer Adults during the Early Days of the COVID-19 Pandemic

**DOI:** 10.3390/ijerph182412919

**Published:** 2021-12-08

**Authors:** Pamela Valera, Madelyn Owens, Sarah Malarkey, Nicholas Acuna

**Affiliations:** 1 Rutgers School of Public Health, Piscataway, NJ 08854, USA; mdo55@sph.rutgers.edu (M.O.); shm76@sph.rutgers.edu (S.M.); 2 Department of Population and Public Health Sciences, Keck School of Medicine, University of Southern California, Los Angeles, CA 90032, USA; nacuna@usc.edu

**Keywords:** COVID-19, Queer, LGBT, cigarette smoking, vaping, minority stress

## Abstract

The purpose of this narrative study is to describe the vaping and smoking characteristics of Queer people ages 18–34 before March of 2020 and to better understand how the COVID-19 pandemic has impacted those behaviors since March of 2020. In total, 31 participants were screened. Thirteen participants were screened prior to the emergence of COVID-19, and 18 were screened when study protocols transitioned to a remote setting (pre and during). Of the 27 eligible participants, a total of 25 participants completed the study. Most participants (*n* = 13) self-identified as male, followed by five identified as female, four self-identified as gender non-binary, and three identified as transgender. The most common sexual orientation amongst participants was gay (*n* = 10), with bisexual being the second-most reported. Approximately 20 Queer participants reported using cigarettes, 14 participants self-reported using electronic devices, and 11 reported using hookah. Twenty participants reported smoking ten or less, and four self-reported using 11–20 cigarettes per day. Approximately, 92% of participants (*n* = 23) indicate that they are using an e-cigarette and regular cigarettes, and 57% of participants (*n* = 12) report using one pod or cartridge per day. The three themes that emerged in this study are: (1) Queer people during COVID-19 are experiencing heightened minority stress; (2) Queer people are unfamiliar with smoking cessation; and (3) vaping and smoking are attributed to stress and anxiety. Queer participants are likely to be dual users of cigarette and vaping products. This present study provides increasing evidence that Queer people are experiencing heightened stress and anxiety and using cigarette smoking and vaping to cope during the COVID-19 pandemic.

## 1. Introduction

Smoking tobacco products (e.g., cigarettes, cigars, loose tobacco, kreteks) is the leading preventable cause of death, disease, and disability in society [1]. For this study, Queer is used as “…an umbrella term for anyone who is not heterosexual or cisgender…” [2]. Studies have shown that Queer adults have an increased risk of smoking cigarettes and vaping products than their heterosexual counterparts [1,3,4,5]. Queer adults smoke tobacco products at significantly higher rates (ranging from 33% to 45%) compared to heterosexual adults (14%) [1,3,4,5]. Similar to cigarette smoking rates, Queer people are five times more likely to use e-cigarettes or vaping products than the general adult population [4,5,6,7]. Differences among smoking and vaping prevalence between Queer sub-populations have been supported by another study that explored transgender women seeking hormone replacement therapy [8]. This study suggests a prevalence rate of smoking at 43% [8]. Moreover, Queer adults use these products in combination with alcohol and other substances [5,6,7].

Prevalence data is based on studies that use population-based surveys and observational studies to understand smoking behaviors and tobacco products across a sample of Queer groups [8,9,10,11]. The Population Assessment of Tobacco and Health (PATH) Study, a national longitudinal study of tobacco use and its impacts on people in the US, [12] describes the lifetime use of tobacco products by using two measures: sexual orientation and six types of tobacco products [5]. Researchers analyzing PATH data have reported differences in tobacco and vape product use rates among Queer sub-populations [4]. Jamal and colleagues [13] found that sexual orientation other than heterosexual (i.e., identifying as LGB, other, or questioning) was associated with an increased likelihood of cigarette smoking. The 2015 US Transgender Survey (USTS) is the only national dataset to include psychosocial variables, substance use, discrimination, and smoking behaviors among 27,715 transgender people [14]. Results from the USTS found that trans and gender-nonconforming people who report dual-use (use of e-cigarettes and cigarette smoking) and experiences of discrimination (i.e., unequal treatment, verbal harassment, or physical assault) significantly increase the odds for e-cigarette use, cigarette smoking, and dual-use of these products [14].

Tobacco smoking has been associated with adverse disease, and extensive data point to the negative impact of smoking tobacco and vaping on lung and respiratory health [1,15]. Cumulative exposure to cigarette smoke is an independent risk factor for hospital admission and death from COVID-19. Heavy smokers are more than two times more likely to be hospitalized for COVID-19 and almost two times more likely than never-smokers to die from COVID-19 [15,16]. However, little is known about the ways COVID-19 is impacting Queer people in early adulthood who smoke cigarettes and vape. Given the gap in the evidence, this qualitative study describes the smoking behaviors and vape use among Queer people.

The purpose of this current study is to describe the vaping and smoking behaviors of Queer people in early adulthood before March of 2020 and to develop a better understanding of how the COVID-19 pandemic has impacted those behaviors since March of 2020. The research question of this study was: What is the lived experience of Queer people who smoke cigarettes and use vape products before and during the COVID-19 pandemic?

## 2. Methods

### 2.1. Qualitative Approach and Research Paradigm

Narrative methods approach and interpretive research design were used to structure this qualitative research study [16,17,18] given the chronological unfolding of COVID-19 and how the pandemic intersected with how study participants experience tobacco smoking and vaping. Creswell and Poth [19] explain that in the narrative approach, events are presented as non-random. There is some form of chronology and movement through time and space. Stories are told by individuals, and these experiences are expressed in the lived and told stories of participants. Another critical component of narrative research is restory [16]. After capturing their stories, the research team organized aspects of the participants’ lived experiences according to common themes using an interpretivist approach, which involves employing multiple methods (e.g., memoing) to reflect and make meaning of different aspects and experiences of Queer participants who smoke cigarettes and use vape products before and during the COVID-19 pandemic [20].

Collaboration is the third element of the narrative approach where the researcher records participants’ stories, and the participants are provided space to share their stories. Both individuals are involved in the process of change [21]. Given that Queer people are an underrepresented population in research, the narrative approach was also chosen to elevate the voices, stories, and experiences of people less often heard.

### 2.2. Reflexivity and Researcher’s Characteristics

Reflexivity and the researchers’ characteristics are important in establishing rigor in the study of the experiences of Queer smokers and vape users. The interviewers were cisgender master’s level and one doctoral student cisgender public health students who reflected the racial, ethnic, and age diversity of the study participants but did not identify as Queer or smoke cigarettes or vape products. However, for the study’s data analysis, researchers’ social position reflected the study participants in terms of age, personal experience, gender, and sexual orientation. This approach served to study an unfamiliar experience among the interviewers, allowing participants to be the experts of their story, while researchers analyzing the data permitted the co-construction of the narrative as told by the study participants and interpreted by the researchers [22,23].

### 2.3. Ethical Issues Pertaining to Human Subjects

This study received IRB ethical approval through Rutgers University, and pseudonyms were used to protect the identity and confidentiality of the study participants.

### 2.4. Context and Data Collection Methods

**Figure 1:**
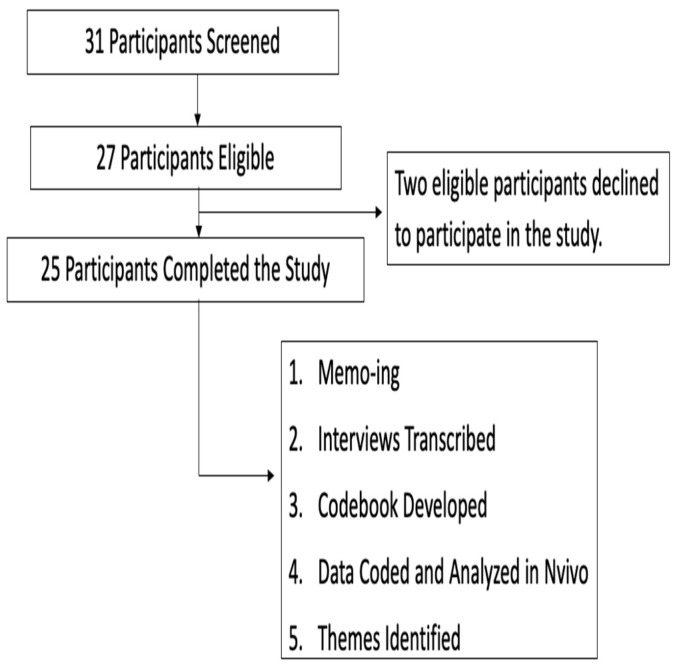
Data collection flowchart.

Before the COVID-19 pandemic emerged, recruitment flyers were distributed digitally and physically to various community organizations that serviced the Queer population throughout the Greater Newark and New Jersey areas. During COVID-19, data collection transitioned to a virtual setting requiring changing the recruitment process. The research flyers were distributed solely through social media platforms (e.g., Instagram, Facebook, and Twitter). Potential participants completed a screener delivered via telephone prior to conducting the interviews to be eligible to participate in the study. When recruitment became remote, the screener and semi-structured interviews were conducted using Microsoft Teams video, approved by Rutgers University Institutional Review Board (IRB). Figure 1 describes the data collection process.

### 2.5. Data Collection, Instruments, and Technologies

The data collection instruments used in the study included a screener, interview protocol, memos, a brief smoking questionnaire, Microsoft Teams for interviewing study participants remotely, and NVivo qualitative software for managing and analyzing the data.

Screener

The screener contained a total of 11 questions that collected basic demographic information from potential participants (e.g., state of residence, sexual orientation, age). The screener was used to determine whether participants were eligible to participate in the study. The eligibility criteria included: (1) identify as lesbian, gay, bisexual, or transgender; (2) between the ages of 18–34; (3) able to speak, read, and write in English well enough to understand consent procedures; (4) smoked at least five cigarettes per day over the past seven days, as confirmed by self-report; (5) current e-cigarette or vaping use; and (6) a willingness to take part in a semi-structured interview.

Semi-Structured Interview Guide

Before COVID-19, the interview guide facilitated a conversation with participants discussing an overview of their smoking and vaping behaviors, smoking habits related to their Queer identity, and smoking cessation treatment. Examples of the interview questions were: “tell me about the first time you tried a cigarette? (probe: who gave it to you, age, describe the experience).” “Describe your smoking or vaping routine (probe: how often, how much do you spend per week).” The interview questions were piloted to two Queer college students to improve the interview guide.

After data collection transitioned to a remote setting, the interview guide also incorporated questions related to COVID-19 and smoking (e.g., how the pandemic has affected participants’ smoking habits). A few examples of the interview questions were: “How are you dealing with the coronavirus threat?” “How has this whole coronavirus outbreak affected the way you feel about smoking/vaping in general?”

Smoking Questionnaire

In addition to the interview guide, study staff also went through a questionnaire with participants, which assessed their medical and psychological history, history of substance use, previous quit attempts, and smoking behavior and history.

Memoing

After each semi-structured interview, interviewers wrote memos about each interview, reflecting on ideas by extrapolating patterns and concepts that the participant shared with them during their time together [15]. This experience of memoing enabled the interviewer to have an intense relationship with the data.

The qualitative semi-structured interviews lasted approximately 45 min to 1 hr. for those who completed the study in person and 1 hr. to one and half hours for those who were interviewed remotely. Participants who completed the study were compensated USD 50 in cash (before COVID-19) or a USD 50 online Amazon gift card (during COVID-19).

Technologies

Microsoft Teams was used to interview study participants remotely and all the interviews were digitally recorded with permission from every participant provided by signing the informed consent document. NVivo was used to manage and analyze the data.

### 2.6. Qualitative Sampling

In-depth semi-structured interviews among Queer adults between the ages of 18 to 34, currently use vaping products and smoke cigarettes were conducted between September 2019 and September 2020.

A purposive sample of 31 participants was recruited; however, only 27 were eligible to participate.

### 2.7. Data Processing

The interviews were digitally recorded and uploaded into NVivo software [24] for transcribing, coding, data management, and determining inter-coder reliability. The digital recordings were transcribed by QSR automated transcription software, and members of the research team reviewed each transcript for accuracy and de-identified the data by deleting information that would allow others to identify the study participants. The team reviewed the transcriptions to clean and ensure quality, and memos were also uploaded and coded in NVivo. A thematic reflexive analysis allows researchers to derive meaning across study codes and subgroups to identify themes [24,25,26].

### 2.8. Data Analysis

#### Codebook

The codebook was developed using memos and interview data to develop relevant categories and patterns (Table 1). These patterns were then used to develop primary codes, secondary codes, and tertiary codes. In response to the COVID-19 pandemic, additional codes were added to capture data related to how Queer people responded to the pandemic. Finally, during the development of the codebook, codes were reevaluated, and some were altered to incorporate the lived experience being shared by study participants.

### 2.9. Inter-Coder Reliability

Inter-coder reliability was established by two research team members independently using the same codebook developed to code five transcripts at random. The coding comparison query in NVivo enabled the coders to compare their coding to measure inter-coder reliability. The degree of agreement between the two coders were calculated to determine the percentage of agreement using the Kappa coefficient formula. The agreement between the two coders was above 70% except for the code social determinants of health. The codes with the highest amount of agreement were COVID-19, vaping history, and smoking history, with 97.3% agreement. Discrepancies with the coding process were discussed with the research team to ensure the quality of the coding process.

### 2.10. Establishing Rigor and Trustworthiness of Data

Several approaches were used to establish the rigor and trustworthiness of the data. These were: (1) team members re-read the transcripts multiple times; (2) interviewers reflected on the interviews by memoing their experience; (3) coding and recoding of the data; (4) the process inter-coding reliability between two coders; and (5) using reflexive thematic analysis and interpretive design to develop the themes of the study.

### 2.11. Qualitative Analysis

NVivo was used to develop an audit trial of the materials and memos collected to explore the research question and identify assumptions that researchers may hold about the topic. The first author created a visual map during the analysis to develop the themes after the coding process was complete.

Reflexive thematic analysis is a qualitative analytical approach in which stories about a phenomenon are collected from study participants and the context and structure of these stories are analyzed by the researchers to answer a research question [18,19,20,21,22,23]. Whether through semi-structured interviews, focus groups, memos, or other data collection processes, the researchers reflected on what and how a story was shared across different data sets to identify patterns and meaning. The research team extracted the following codes from NVivo: COVID-19, Smoking Cessation Treatment, Smoking History, Vaping History, and Illicit Drug Use to generate the themes.

The three primary themes that were identified from both the interview data and memos were: (1) Queer people during COVID-19 are experiencing heightened minority stress; (2) Queer people are unfamiliar with smoking cessation; and (3) vaping and smoking are attributed to stress and anxiety.

## 3. Findings

Of the 27 eligible participants, a total of 25 participants completed the study. Most of the participants who completed the study (*n* = 13) self-identified as male, followed by five identified as female, four identified as gender non-binary, and three identified as transgender. A majority of participants enrolled in the study were African American (*n* = 13). The most common sexual orientation amongst participants was gay (*n* = 10), with bisexual being the second-most reported. Only one participant identified as lesbian (the lower proportion of study participants identified as a lesbian is further discussed in the limitations section). Table 2 describes the participants’ demographic characteristics, and Table 3 provides information about the participant’s gender, sexuality, and employment status.

The smoking and vaping behaviors among participants varied. Twenty participants reported using cigarettes, 14 participants self-reported using electronic devices, and 11 self-reported using hookah. Only two participants (8%) reported using large cigars. Participants were able to check multiple types of tobacco or smoking device. The number of cigarettes participants reported smoking per day also varied. The majority of participants (*n* = 20) reported smoking ten or less while four reported using 11–20 cigarettes per day. At least 32% (*n* = 8) of the participants smoke their first cigarette more than 60 min after waking up and between 5–30 min after waking up (*n* = 8). Moreover, 92% of participants (*n* = 23) indicated that they are using an e-cigarette, and 57% of participants (*n* = 12) reported using one pod or cartridge per day. Table 4 describes smoking behaviors, and Table 5 describes vaping behaviors of study participants.

Theme One: Queer people during COVID-19 are experiencing heightened minority stress

COVID-19 has disproportionally impacted the lives of Queer people. All the participants interviewed during the early days of the pandemic noted how they were in a major transition in their lives with much uncertainty, causing stress and anxiety. For example, one participant who is non-binary and gay said:

“*Life is a transition right now. Like, I wake up every day and am like, what is going on? Like, I need to, you know, [should I] cancel my car insurance, do I get room furniture? I think my brain has been in this space a lot of the pandemic, it’s been in survival mode. What day is it? It’s Wednesday. Yeah, I’m already on my second pack, so it’s probably going to be like a three-pack week for me*.”

Another non-binary participant who is Queer indicated the following:

“*It has made me feel more urgency around smoking or at least more anxiety. I still haven’t been able to overcome the actual psychological addiction*.”

A cisgender male participant who is gay experienced a lot of anxiety, he noted:

“*and at the beginning, I was freaking out. I needed a friend. I didn’t have my friends, [they] were like, busy as hell. And they were hiding from it too. And I was just; it was dark*.”

### 3.1. Theme Two: Queer People Are Unfamiliar with Smoking Cessation

Regardless of which period they participated in the study, all study participants agreed that tobacco smoking is not good for one’s health. While participants recognized the health complications with COVID-19 and smoking, participants did not understand smoking cessation. For instance, several cisgender men who identify as gay did not know what smoking cessation meant:

Interviewer: So, what does cessation mean when I say smoking cessation?
CH1005: Cessation…No, I don’t know cessation.
Interviewer: So, the first thing I want to ask is, do you know what smoking cessation is?
CH1011: Is that the feeling you get when you are smoking?
Interviewer: So, cessation, not sensation. Smoking cessation.
CH1011: Cessation. Oh. OK. So, I have to say. So, no, I don’t know what that is.

Participants often confused smoking cessation with sensation, assuming it is a “feeling” after smoking a cigarette. Furthermore, another cisgender and gay participant indicated that there are few resources to help Queer people stop smoking. He noted the following:

*There’s not a lot of resources out there to help people stop smoking in the LGBT community. The only time you really hear the LGBT sector to tell people to stop smoking is when you are dealing with HIV. So, like that’s the only time I ever really see doctors, or anybody really, talk about smoking with the LGBT community*.

### 3.2. Theme Three: Vaping and Smoking Are Used to Lessen Heightened Stress and Anxiety

Experiencing daily accounts of stress and anxiety may trigger using a vape device or a need to smoke a cigarette. This is particularly evident during the early days of the pandemic when there were a lot of mixed messages about public safety protocols. Several Queer participants used cigarettes or vaping to reduce stress. One gay cisgender male stated the following:

*Cigarettes is really a stress reliever for me personally. That’s mostly why I’m really smoking. You’ll see me smoking cigarettes, if I’m at work stressed, you’ll see me in the back vaping cause when I am angry upset*.

A transgender woman noted that:

*Smoking is very bad. We know that. We see the commercials. We see the surgeon general warnings in the bottom of the cigarette packs. But do we consider them? No, because in that moment we are stressed, or we are going through something*.

Lastly, a participant who is bisexual smokes cigarettes to reduce anxiety especially during sex work. They indicated the following:

*Especially when it comes down to like sex work in the LGBT community. It’s definitely, always something that helps keep that anxiety down while you on the. It’s…there’s a certain amount of anxiety with being LGBT and in space. Will, I be assaulted because I’m out here on the street? And I’m not passing. You know*.

Stress and anxiety are a common part of life for Queer people. However, COVID-19 has heightened Queer peoples’ stress, bringing new challenges to address [17,27].

## 4. Discussion

This study explored the lived experience of Queer people who smoke cigarettes and vape prior to—and during the early days of—the COVID-19 pandemic. In this study, Queer participants are likely to be dual users of cigarette and vaping products. Furthermore, these findings provide increasing evidence that Queer people are experiencing heightened stress and anxiety. Most study participants report an increase in their smoking and vaping behaviors to manage stress and anxiety. Queer participants also experienced social isolation due to social distancing guidelines during the early days of the pandemic. While this impact is not directly related to smoking or vaping, it is essential to consider that social connectedness—especially among Queer communities—acts as a protective factor to other adverse health outcomes [28,29].

Interestingly, some participants reported combining marijuana products when smoking tobacco products or using vape products. Given the increased risk associated with smoking and vaping behaviors, [15] the results from qualitative studies are critical when attempting to understand the ways that the COVID-19 pandemic has increased tobacco-related health disparities from the lived experiences of Queer people. Additional research is required to understand polysubstance use in Queer people [11,14].

*Strengths and Limitations*

There are several notable strengths of the current study. This study includes a rigorous qualitative method such as coding and recoding, achieving intercoder reliability, developing the codebook, using narrative research methods, memoing, and using reflexive thematic analysis to co-create the meaning behind tobacco smoking and vaping in Queer people. Other important strengths include an expansive sample of diverse Queer study participants.

However, some limitations need to be considered when interpreting the findings. First, women who have sex with women but reported “not having sex within the past six months” were excluded. Given the social distancing guidelines present during data collection, this is likely the reason why only one participant identified as lesbian. Second, methods to capture data about participants with intersecting gender identities (i.e., trans and gender-nonconforming, or male and transgender) were not incorporated into the study design. Therefore, it is likely that the number of trans-identified study participants may be underreported. Third, Queer people with intellectual and developmental disabilities were not included due to restrictions of consent required to participate in the study. Fourth, due to the unknown sexual and gender positionality of interviewers that collected the data, and the sensitive nature of disclosing Queer identity, researchers’ bias may have been introduced. Finally, due to the positionality of those analyzing the research as insiders, bias may have been introduced as well. Future research with Queer smokers and vape users may be able to overcome this limitation by including a more expansive and diverse population and by using member checking, the process validating the study participants’ responses [30].

*Implications*

There are several implications for future work and research. Targeted resources to help Queer people quit smoking and vaping and address anxiety and minority stress are needed, particularly during the COVID-19 pandemic. When asked about cessation resources in the community, participants shared that they were not aware of any Queer-specific cessation resources. Second, when reporting research about LGBTQ+ people “Sexual and Gender Minorities” is sometimes used as an umbrella term to describe multiple subpopulations that make up the LGBTQ+ community. However, LGBTQ+ sub-populations use terms such as “gays”, “queers”, “LGBTQ+ people”, and others as umbrella terms. When conducting future research with LGBTQ+ people we recommend working with study participants to identify language that most accurately represents those participating in the research. It is also important to carefully consider the positionality of those reporting the research, considering that Queer at one point was used as a derogatory slur and has since been reclaimed by Queer activists. Third, additional research about Queer people in general and underrepresented Queer sub-populations is needed (i.e., Queer people who do not speak English, are Non-binary, Agender, Genderqueer, and Queers with Intellectual and Developmental Disabilities). Insiders should lead future research and incorporate an intersectional approach, focusing on specific subpopulations most excluded from research studies.

## 5. Conclusions

The COVID-19 pandemic does not impact all people equally. Health outcomes are impacted by social determinants of health, including current environmental factors where we all live, work, and play. Evidence that elevates the voice of people with lived experience navigating intersecting oppressions is required to help equip public health experts to make equitable, just, and informed decisions regarding how to respond to the current pandemic and prevent future ones.

## Figures and Tables

**Table 1:** Queer smoking cessation code book.

First Code	Second Code	Third Code	Inclusion	Exclusion	Example
COVID-19	Changing Behavior Coping with COVID-19 Threat Impact of COVID-19 on Smoking and Vaping Information About COVID-19 Knowledge of COVID-19 People at Risk for COVID-19 Sentiments About COVID-19	Others in Community Self Social Distancing News and Social Media Social Networks Origin of COVID-19	Any discussion of COVID-19.	No mention of changing behavior, coping, impact on smoking and vaping, COVID-19 information or knowledge, people at risk, and sentiments about COVID-19,	*“I have cut down my smoking since the pandemic because of the droplets that could be coming out [when smoking] … I don’t want to be the ignorant person to infect someone.”*
Preference over e-cigarettes vs. cigarettes	Culture of LGBT Community Desire to Appear More Masculine Social Norms		Any discussion about the differences between English COVID-19 search results compared to Spanish COVID-19 search results. Any discussion about why a participant prefers one type of product over another.	No mention about why a participant prefers one type of product over another.	*“The thing about electronic cigarettes is that the electricity is just something different about it flowing through my body. One time I picked up an electronic cigarette and it really shocked me in my mouth … I’ll just stick to what I know.”*
Routine	Smoking Vaping		Any mention of routines related to smoking or vaping.	No mention of routines related to smoking or vaping.	*“I only smoke more when I’m stressed, or if I’m drinking … The most cigarettes I have smoked in a day now is about five to six.”*
Smoking Cessation Treatment	Barriers to Smoking Cessation Treatment FDA Approved Medication Experience with Smoking Cessation Programs Knowledge of Smoking Cessation LGBT Smoking Cessation Treatment Steps to Quitting	NJ Quit Line Quit Center	Any mention of smoking cessation, barriers to quitting, experience with smoking cessation programs.	No mention of smoking cessation, barriers to quitting, experience with smoking cessation programs.	*“I had a roommate that had a heavy smoking problem. He actually got a podcast of self-help to kind of talk him through not smoking. He said those are really helpful, too. So, if I was trying to do something long term, I might incorporate something like that as well. I think that gum would be something I would use.”*
Smoking History	Benefits of Smoking Provider Discussion Reasons for Smoking		Any mention about benefits of smoking, provider discussing smoking behavior with study participant, motivations for smoking	No mention of benefits of smoking, provider discussing smoking behavior with study participant, motivations for smoking	*“I remember vaguely my first time I actually smoked a cigarette. I was in high school. I think I was about 15 or 16. The young lady who lived next door to me, she was a smoker. So I would see her smoke and I thought it was cool.”*
Smoking or Vaping Identity			Any mention of tobacco smoking and vaping as part of their self-identity, culture, motivations, etc.	No mention of cigarette smoking and vaping as part their self-identity, culture, motivations, etc.	*“I think that I am a social smoker. Although, even when I’m not in the social environment, if I do get by myself every now and then, I’ll have one by myself.”*
Social Determinants of Health	Health Problems Minority Stress Stigma and Discrimination		Any discussion about health problems, minority stress, and stigma and discrimination.	No mention of health problems, minority stress, and stigma and discrimination.	*“…most of the issues come from people dealing with housing, or dealing with their own personal issues that they have … they’re looking for a way out. Drugs, cigarettes, all of these things are their way out.”*
Thoughts on Vaping Crisis			Any mention of vaping crisis.	No mention of vaping crisis.	*“I stopped smoking vapes after hearing about everybody with the lung diseases…”*
Tobacco Products	Money Spent		Any discussion about tobacco products currently being used, previously used, and how much is spent acquiring tobacco products.	No mention of tobacco products currently being used, previously used, and how much is spent acquiring tobacco products.	*“I tend to order a lot of cigars. I order in bulk…I would say probably a couple of packs a week.”*
Vaping History	Benefits of VapingMoney Spent on e-cigs. Reasons for Vaping Risk of Vaping Vaping Devise		Any discussion about previous vaping behavior, perceived benefits from vaping, money used to acquire vaping products, motivations for vaping, perceived risks of vaping, and any conversations about vaping device.	No mention of previous vaping behavior, perceived benefits from vaping, money used to acquire vaping products, motivations for vaping, perceived risks of vaping, and any conversations about vaping device.	*“First time I tried to vape was in D.C. It was OK. It really didn’t work for me because of the simple fact that it’s not as strong…”*
Drug Use	cannabis, tetrahydrocannabinol (THC)-containing e-cigarette or other substance		Any discussions about vaping cannabis, THC or other substances in vaping device	No mention of cannabis, THC or other substances in vaping device	*“…once they were able to start putting THC into [vapes] that’s why everybody wanted to vape.”*

**Table 2:** Demographics of participants (*n* = 25).

Variable	*n* (%)
Age	
18–24	9 (36.0)
25–29	7 (28.0)
30–34	9 (36.0)
Race	
Native American or Alaskan Native	1 (4.0)
Asian	2 (8.0)
Black or African American	13 (52.0)
White	8 (32.0)
Two or More Races	1 (4.0)
Are you Hispanic or Latino	
Yes	2 (8.0)
No	23 (92.0)
Highest Educational Attainment	
High School or GED	4 (16.0)
Some College/Technical School	10 (40.0)
Bachelor’s Degree	8 (32.0)
Graduate Degree	3 (12.0)

**Table 3:** Gender, sexuality, and employment demographics (*n* = 25).

Variable	*n* (%)
Current Gender	
Female	5 (20.0)
Male	13 (52.0)
Transgender	3 (12.0)
Gender non-binary	4 (16.0)
Sexuality	
Bisexual	7 (28.0)
Gay	10 (40.0)
Heterosexual	1 (4.0)
Lesbian	1 (4.0)
Pansexual	2 (8.0)
Queer	4 (16.0)
Employment Status	
Employed	13 (52.0)
Unemployed	6 (24.0)
Homemaker/caretaker	1 (4.0)
Student	5 (20.0)

**Table 4:** Smoking behaviors (*n* = 25).

Variable	*n* (%)
Tobacco Products Used ^a^	
Cigarettes	20 (80.0)
Little Cigar with filter	7 (28.0)
Little Cigar without filter	3 (12.0)
Large Cigar	2 (8.0)
Cigarillos	6 (24.0)
Electronic devices	14 (56.0)
Hookah	11 (44.0)
Cigarettes Smoked Per Day ^b^	
10 or less	20 (80.0)
11–20	4 (16.0)
21–30	0 (0)
31 or more	0 (0)
Time to first Cigarette After Waking Up ^b^	
More than 60 min	8 (32.0)
31–60 min	1 (4.0)
5–30 min	8 (32.0)
Less than 5 min	4 (16.0)

^a.^ Study participants could select multiple choices. ^b.^ Missing data.

**Table 5:** Vaping behaviors (*n* = 25).

Variable	*n* (%)
Have you ever used an e-cigarette?	
Yes	23 (92.0)
No	2 (8.0)
Do you use e-cigarettes to quit tobacco? ^a^	
Yes	11 (44.0)
No	12 (48.0)
How many pods/cartridges do you use per day? ^a^	
Zero	1 (4.8)
Less than one	2 (9.5)
One	12 (57.1)
Between one and two	1 (4.8)
Two	1 (4.8)
More than two	1 (4.8)
Unknown	3 (14.3)

^a.^ Missing data.

## Data Availability

The data presented in this study are available on request from the corresponding author. The data are not publicly available to retain participant privacy.
